# How low can you go? Short-read polishing of Oxford Nanopore bacterial genome assemblies

**DOI:** 10.1099/mgen.0.001254

**Published:** 2024-06-04

**Authors:** George Bouras, Louise M. Judd, Robert A. Edwards, Sarah Vreugde, Timothy P. Stinear, Ryan R. Wick

**Affiliations:** 1 Adelaide Medical School, Faculty of Health and Medical Sciences, The University of Adelaide, Adelaide, Australia; 2 The Department of Surgery – Otolaryngology Head and Neck Surgery, University of Adelaide and the Basil Hetzel Institute for Translational Health Research, Central Adelaide Local Health Network, South Australia, Australia; 3 Department of Microbiology and Immunology, University of Melbourne at the Peter Doherty Institute for Infection and Immunity, Melbourne, Australia; 4 Flinders Accelerator for Microbiome Exploration, College of Science and Engineering, Flinders University, Adelaide, Australia

**Keywords:** assembly polishing, bacterial genome assembly, genome polishing, Oxford nanopore sequencing

## Abstract

It is now possible to assemble near-perfect bacterial genomes using Oxford Nanopore Technologies (ONT) long reads, but short-read polishing is usually required for perfection. However, the effect of short-read depth on polishing performance is not well understood. Here, we introduce Pypolca (with default and careful parameters) and Polypolish v0.6.0 (with a new careful parameter). We then show that: (1) all polishers other than Pypolca-careful, Polypolish-default and Polypolish-careful commonly introduce false-positive errors at low read depth; (2) most of the benefit of short-read polishing occurs by 25× depth; (3) Polypolish-careful almost never introduces false-positive errors at any depth; and (4) Pypolca-careful is the single most effective polisher. Overall, we recommend the following polishing strategies: Polypolish-careful alone when depth is very low (<5×), Polypolish-careful and Pypolca-careful when depth is low (5–25×), and Polypolish-default and Pypolca-careful when depth is sufficient (>25×).

**Impact Statement**

Oxford Nanopore Technologies sequencing data can be used to generate near-perfect bacterial genomes. However, when perfect genomes are required, it is necessary to also perform ‘polishing’, which uses data from complementary sequencing platforms to fix remaining errors. When polishing a genome, it is important to avoid introducing new errors which may make the genome sequence worse. In this study, we introduce two polishing software tools, Pypolca and Polypolish v0.6.0, both of which are designed to carefully fix errors in genomes without introducing new errors. We benchmark these tools, along with other published polishing tools, using different quantities of sequencing data. The results of this study provide clear recommendations: which polishing tools to use and how much sequencing data is required for the best results. This information will help researchers produce higher quality bacterial genome sequences, making truly error-free genomes easier than before.

## Data Summary

Pypolca is open-source and freely available on Bioconda, PyPI and GitHub (github.com/gbouras13/pypolca). Polypolish is open-source and freely available on Bioconda and GitHub (github.com/rrwick/Polypolish). All code and data required to reproduce analyses and figures are available at github.com/gbouras13/depth_vs_polishing_analysis. All FASTQ sequencing reads are available at BioProject PRJNA1042815. A detailed list of accessions can be found in Table S1, available in the online version of this article.

## Introduction

Recent advances in the accuracy of Oxford Nanopore Technologies (ONT) sequencing have made it possible to recover near-perfect complete bacterial genomes using only ONT long-read sets. Remaining errors are often fewer than ten per genome [1] and usually arise in long homopolymer and DNA modification sites that are difficult to resolve with current ONT data [2,3]. Therefore, using short reads to polish ONT-only assemblies still provides accuracy benefits [4,5]. When polishing near-perfect genomes, the burden shifts from short-read polishers resolving as many errors as possible (i.e. reducing false negatives), to ensuring that polishers do not introduce new errors (i.e. reducing false positives).

For example, consider a hypothetical polisher that fixes 100 % of errors in a given genome but also introduces ten new errors. For an assembly with hundreds of errors (e.g. an ONT-only Trycycler [6] assembly in 2021), this polisher would greatly improve accuracy. However, for a near-perfect genome with only five errors (e.g. an ONT-only Trycycler assembly in 2024), this polisher would introduce more errors than it fixed.

Given the lack of errors remaining in current ONT-only assemblies, another consideration is the short-read depth required for polishing. While 30× coverage is the standard for variant calling in the context of human genomics [7], the relationship between polishing and sequencing depth in bacterial contexts has not been investigated.

In this study, we present three advances. Firstly, we introduce Pypolca, a Python re-implementation of POLCA [8] with added features. Secondly, we introduce a new version of Polypolish [9] (v0.6.0). Both Pypolca and Polypolish v0.6.0 include a new ‘--careful’ option that reduces false positives. Finally, we investigate how the performance of Pypolca, Polypolish and other short-read polishing tools vary with short-read depth using a panel of nine deeply sequenced bacterial strains with near-perfect Trycycler assemblies [6]. We show that approximately 25× genome coverage is sufficient to polish almost all ONT-only assembly errors, and that at very low depths (<5×), all short-read polishers other than Pypolca with ‘--careful’ and Polypolish commonly introduce errors.

## Methods

### Pypolca

Pypolca is a Python-based re-implementation of the short-read polisher POLCA [8]. In comparison to POLCA, Pypolca implements a simplified command-line interface, allowing the user to clearly specify input and output files. Additionally, it is available and installable on both macOS and Linux (whereas POLCA can only be run on Linux) and does not require the installation of the MaSuRCA genome assembler like POLCA [10].

**Fig. 1.**
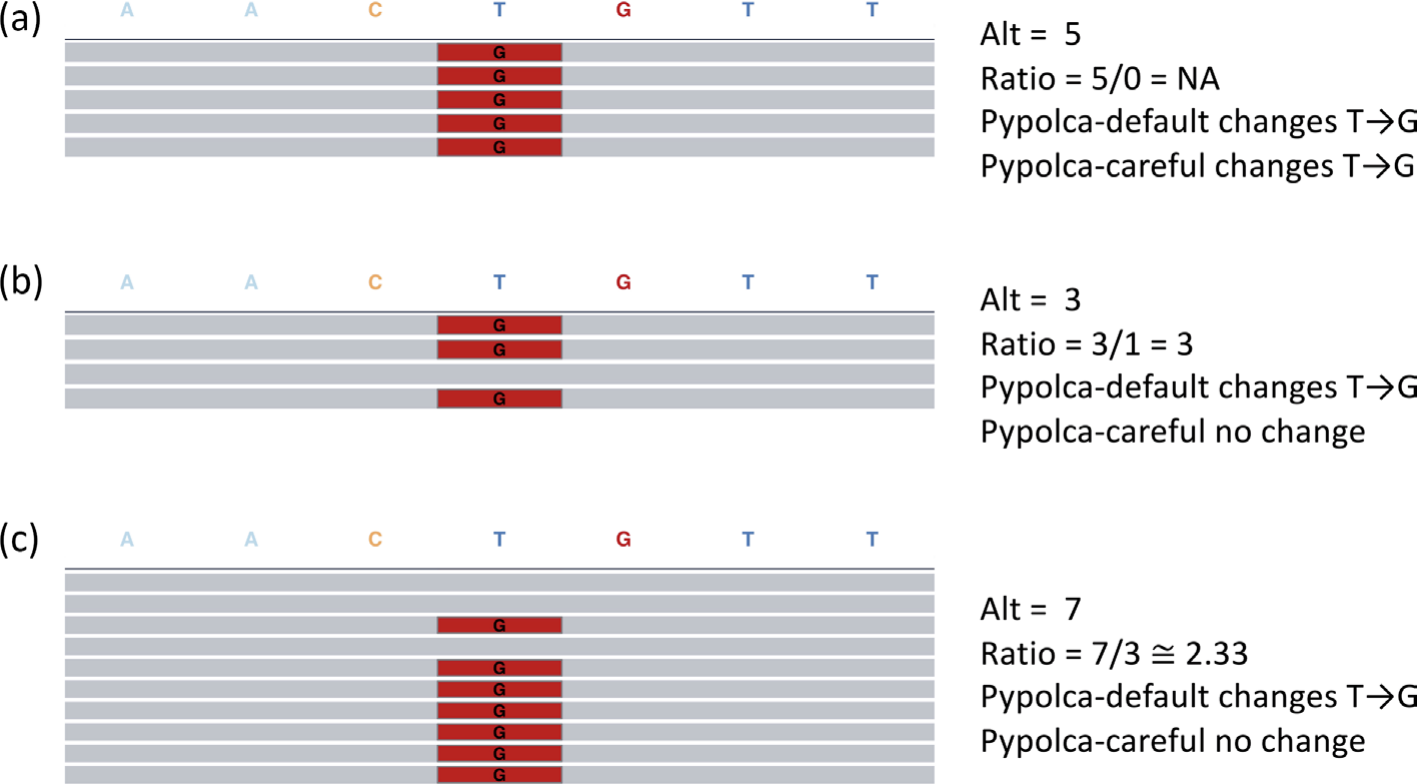
Pypolca polishing decisions at low sequencing depths. Each pileup plot shows simulated reads (represented by horizontal grey bars) aligned to an assembly sequence AACTGTT. Positions that are the same as the assembly are coloured grey while those that differ are coloured red. ‘Alt’ refers to the number of reads aligning to the alternative allele. ‘Ratio’ refers to the number of reads aligning to the alternative allele divided by the number of reads aligning to the assembly allele. Pypolca-default will change all bases where Alt is at least two (‘--min_alt 2’) and Ratio is at least two (‘--min_ratio 2’). Pypolca-careful will change all bases where Alt is at least four (‘--min_alt 4’) and Ratio is at least three (‘--min_ratio 3’). (**a**) All five aligned reads support the alternative ‘G’ (coloured in red) rather than the assembly ‘T’ at the fourth base in the sequence. Both Pypolca-default and Pypolca-careful will change T→G at this position. (**b**) Three aligned reads support ‘G’, while one read supports ‘T’ (grey). In this case, Pypolca-default will change T→G, as at least two reads support the alternative allele and the alternative-to-assembly ratio is greater than two. However, because it has only three supporting reads (under the threshold of four), Pypolca-careful will not change this position and leave it as ‘T’. (**c**) Seven aligned reads support ‘G’ while three support ‘T’. Pypolca-default will change T→G but Pypolca-careful will not, as the ratio between alternative and reference alleles is under the threshold of three.

Pypolca, like POLCA, aligns short reads to an assembly using BWA-MEM [11], processes the alignments with Samtools [12], runs freebayes [13] to call variants between the aligned reads and assembly, and then applies well-supported variants to the assembly. POLCA polishes all variants where: (1) at least two aligned reads support the alternative allele, and (2) there are at least twice as many aligned reads supporting the alterative allele compared to the assembly allele. Pypolca retains these thresholds as defaults (‘Pypolca-default’) but allows users to change condition 1 using ‘--min_alt’ and condition 2 using ‘--min_ratio’. The ‘--careful’ flag sets --min_alt to 4 (at least four reads supporting the alternative allele) and --min_ratio to 3 (at least three times as many reads supporting the alterative allele compared to the assembly allele), which as we show in this study prevents most false positives at low depths without sacrificing error removal. Examples comparing polishing decisions between Pypolca-default and Pypolca-careful are presented in Fig. 1.

### Polypolish v0.6.0

The Polypolish software and algorithm have been described previously [9]. As part of this study, we introduce Polypolish v0.6.0, which is now completely implemented in Rust and adds a ‘--careful’ option for low-depth polishing. The motivation for --careful comes from polishing repeat regions.

Consider a repeat region in a genome that occurs twice but is not completely identical. At low depths of short-reads, it is possible to obtain reads from only one instance of the repeat. In this scenario, Polypolish may ‘polish’ the unsequenced instance of the repeat to be the same as the sequenced instance, thereby introducing a false-positive error.

The --careful flag introduced in v0.6.0 forces Polypolish to ignore all reads with multiple alignments. This makes it unable to fix errors in repeats, slightly reducing its ability to fix errors when short-read depth is high, but it ensures that Polypolish will not introduce false-positive polishing errors in repeat regions. However, Polypolish with default parameters is the only alignment-based method that can consistently fix errors in repeat regions and is therefore useful where short-read depth is high (Fig. S1).

### Sample selection

A panel of nine ATCC bacterial strains were used to compare the effect of depth on short-read polishing of near-perfect (~Q60) ONT-only assembled genomes. The strains were deeply sequenced with 55–528× Illumina depth and 657–3051× ONT depth (Table S1).

- *Salmonella enterica* ATCC 10708
- *Vibrio cholerae* ATCC 14035
- *Vibrio parahaemolyticus* ATCC 17802
- *Listeria ivanovii* ATCC 19119
- *Escherichia coli* ATCC 25922
- *Campylobacter jejuni* ATCC 33560
- *Campylobacter lari* ATCC 35221
- *Listeria welshimeri* ATCC 35897
- *Listeria monocytogenes* ATCC BAA-679

### Sequencing

DNA extraction was performed with the GenFind V2 kit (Beckman Coulter). Illumina library preparation was performed with Illumina DNA Prep using quarter reagents (Illumina). Short-read whole genome sequencing was performed on an Illumina NextSeq 2000 with a 150 bp PE kit (Illumina).

ONT libraries were prepared using the ONT SQK-NBD114-96 and SQK-RBK114-96 kits, and the resultant libraries were sequenced using R10.4.1 MinION flow cells (FLO-MIN114) on a GridION (ONT). ONT data were basecalled using the dna_r10.4.1_e8.2_sup@v4.3.0 model with Dorado v0.5.0.

### Benchmarking

ONT-only assemblies were created using Trycycler v0.5.4 and reoriented to begin with a consistent starting position using Dnaapler [14] v0.7.0 as described by us in previous studies using these data [1,15]. The number of errors in the ONT-only assemblies (compared to our previously published method for generating robust and curated bacterial reference genome assemblies [16]) ranged from 0 to 18 total errors, for a total of 37 errors across all nine genomes. A detailed characterization of all errors can be found in Table S2. Seventeen errors were substitutions, predominantly in *C. lari* ATCC 3 5221 (13/17). Fourteen errors were deletions in homopolymer regions, while one error was a single base insertion. Medaka polishing (v1.11.3, r1041_e82_400bps_sup_v4.3.0 model) was not performed, as we found it to have a net negative impact on accuracy (Table S3).

Short-read FASTQs were first processed with fastp [17] v0.23.4 (default parameters) and then randomly subsampled using the ‘seqtk sample’ command from seqtk [18] v1.4 at 0.1× depth intervals from 0.1× estimated genome coverage to 50×, yielding 500 subsampled short-read sets. In the results, we refer to depths below 5× as ‘very low’, depths from 5 to 25× as ‘low’ and depths above 25× as ‘sufficient’.

For every subsampled read set, the ONT-only assembly was polished using the following polishing tools (using default parameters except where specified):

1. Polypolish v0.6.0 (denoted as ‘Polypolish-default’)
2. Polypolish v0.6.0 with ‘--careful’ (denoted as ‘Polypolish-careful’)
3. Pypolca v0.3.0 (denoted as ‘Pypolca-default’)
4. Pypolca v0.3.0 with ‘--careful’ (denoted as ‘Pypolca-careful’)
5. HyPo [19] v1.0.3
6. FMLRC2 [20] v0.1.8
7. NextPolish [21] v1.4.1
8. Pilon [22] v1.24

Errors were counted by aligning each polished genome to the reference genome, with each substitution counting as one error and each indel counting as one or more errors (defined by the length of the indel). This resulted in 4500 error counts (nine genomes times 500 short-read sets per genome) for each polishing tool, referred to as ‘samples’ in the results. These error counts were then summed across all nine genomes at each depth interval to yield 500 total error counts.

### Optimized parameters for low-depth polishing

For each of the six polishing tools (Polypolish, Pypolca, HyPo, FMLRC2, NextPolish and Pilon), we performed a parameter sweep to optimize for low-depth polishing using the *C. lari* ATCC 35221 ONT-only assembly at 5×, 10×, 15× and 20× short-read depths. We used *C. lari* as it is the smallest genome in this study (required the least time to polish) and had the greatest number of errors (18). Default parameters were first tried, followed by any other parameters recommended in the polisher’s documentation or publication (e.g. ‘eukaryote mode’ for FMLRC2), followed by alternating random parameter sets and modified parameter sets based on the previous best-performing parameter set, until 1000 unique parameter sets were tried. Accuracy was quantified using the sum of remaining errors at each read depth.

### Low-quality draft assemblies

To test the polishing methods on ONT-only assemblies with higher error rates, we generated a Trycycler assembly for each of the nine genomes using fast-basecalled ONT reads (dna_r10.4.1_e8.2_400bps_fast@v4.2.0 model, Dorado v0.5.0). This produced sequences with approximately Q30 accuracy, i.e. about 1000 times more errors than our near-perfect assemblies. We then performed the same read subsampling (500 depth intervals) and polishing (4500 samples per tool) as in our main analysis.

## Results

### Total remaining errors across all nine genomes

**Fig. 2.**
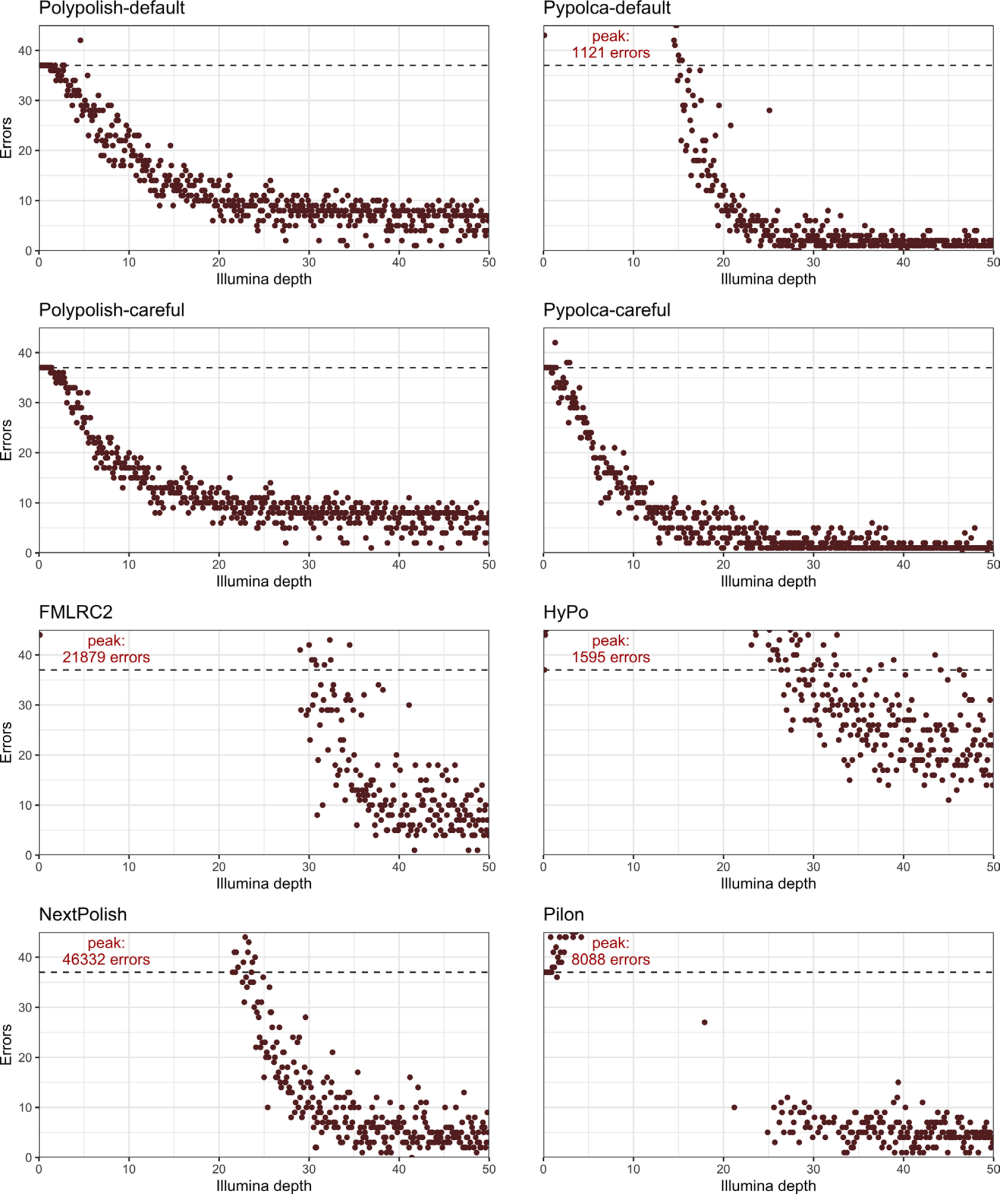
Total errors by depth per polisher. Each plot shows the total number of errors remaining in the nine reference genomes at each interval from 0.1 to 50× depth (*x*-axis) for the eight polishers tested. The dashed lines represent the total Trycycler ONT-only assembly error count of 37 errors. Points above this line indicate that the polisher decreased total accuracy while points below the line indicate increased total accuracy. The *y*-axes for the plots are limited at 45 total errors, with the peak error count labelled in the top left if it exceeds 45. See Fig. S2 for the plots with unrestricted *y*-axes.

Every polisher other than Polypolish-careful increased total errors in at least one interval tested (Figs 2 and S2). Polypolish-default and Pypolca-careful rarely increased total errors (1/500 and 3/500 intervals) and in these cases, the total error counts were only slightly higher (42 total errors for Polypolish-default at 4.6× coverage; 42, 38 and 38 errors for Pypolca-careful at 1.3×, 2.6× and 2.9× coverage, respectively). All other polishers routinely introduced many errors at low depths (Figs 2 and S2). The maximum number of total errors of these polishers ranged from 1121 (Pypolca-default at 3.3× coverage) to 46 332 (NextPolish at 1.6× coverage).

The depths at which these polishers did not decrease overall accuracy varied. The lowest depth (above 2×, below which there may be too few reads for any polishing to occur) that Pypolca-default had 37 or fewer errors was 14.9×, 17.9× for Pilon, 21.5× for NextPolish, 26.4× for HyPo and 29.1× for FMLRC2. The highest coverage where the polishers had 37 or more errors was 15.4× for Pypolca-default, 24.0× for NextPolish, 34.5× for FMLRC2, 43.5× for HyPo and 45.9× for Pilon.

### Changes in per-sample error counts

On a per-sample basis (*n*=4500 for each polisher), Polypolish-careful was the most conservative tool. It never decreased overall accuracy (i.e. increased the total error count compared to the Trycycler ONT-only assembly) in any sample at any interval (Table 1; Fig. S3). It was followed by Pypolca-careful, which decreased overall accuracy in at least one sample in 45/500 depth intervals, in 51/4500 samples overall and never above 16.1× depth, and Polypolish-default (103/500 intervals, 147/4500 samples and never above 21.0× depth). All other polishers commonly decreased overall accuracy in at least one sample in a depth interval, even at higher depths (Table 1; Fig. S3).

**Table 1.** Total number of depth intervals and samples where polishing decreased overall accuracy (i.e. where the total error count was increased compared to the Trycycler assembly) – lower is better

Polisher	Intervals with decreased overall accuracy in at least one sample (*n*=500)	Total samples with decreased overall accuracy (*n*=4500)
FMLRC2	430	2671
HyPo	499	3097
NextPolish	386	2189
Pilon	324	1331
Polypolish-default	103	147
Polypolish-careful	0	0
Pypolca-default	221	1374
Pypolca-careful	45	51

### Polishing performance at sufficient depths

At sufficient depths (above 25×), three polishers (Pypolca-default, Pypolca-careful and NextPolish) had at least one interval where all errors were resolved (Table S4). Every other polisher had at least one interval where only one error was remaining, other than HyPo (minimum of 11 remaining errors). Pypolca-careful had the lowest mean and median error count, followed by Pypolca-default (Table S4). Pypolca-careful had a median error of one, which was a difficult-to-polish error in a long homopolymer on an *S. enterica* plasmid (Fig. S4). Polypolish-default, Polypolish-careful and NextPolish never made ONT-only assemblies worse above 25×, though Polypolish in both modes had significantly lower variance than NextPolish. HyPo, FMLRC2 and Pilon usually (but not always) improved assemblies above 25×, but Pilon sometimes introduced large errors resulting in an overall increase in mean error count.

### Introduced errors at sufficient depths

Introduced errors may be hidden in the above results; for example, if a polisher fixed some errors in an assembly but introduced one error, that would not be apparent from the error counts. We therefore analysed each error separately across the 251 intervals and nine genomes from 25 to 50× depth (Fig. S5). All polishers were commonly able to fix most existing errors, with each polisher fixing at least 36/37 existing errors in at least one interval. However, there were vast differences in the number of errors introduced.

Polypolish-careful introduced only one error across all 2259 samples tested. Polypolish-default introduced seven errors (maximum of one in any sample) and Pypolca-careful introduced 38 errors (maximum of two in any sample). All other polishers commonly introduced errors, ranging from a maximum of 27 in a sample for Pypolca-default to 946 for Pilon. Higher depths generally resulted in fewer introduced errors for FMLRC2, HyPo and NextPolish.

### Types of remaining errors

To determine which types of errors polishers introduced or were unable to fix, each error was characterized either as a substitution, insertion, deletion or mixed (Fig. S6). Errors were denoted as mixed if it belonged to a genomic region with multiple errors containing a combination of substitutions, insertions or deletions. Both Polypolish-default and Polypolish-careful commonly fixed all substitution errors, but often left deletion errors uncorrected. Pypolca in both modes performed well across all error types. FMLRC2 commonly introduced mixed errors while Pilon frequently introduced deletions. HyPo and NextPolish tended to introduce substitutions.

### Combinations of Pypolca and Polypolish

Due to the strong and consistent performance of Pypolca and Polypolish, we compared pairwise combinations of Pypolca-default, Pypolca-careful, Polypolish-default and Polypolish-careful run sequentially. Overall, we found the results to be concordant with the single-tool polishing results, with combinations including Pypolca-careful performing the best overall. The order of polishers had no impact on the results (Table S5; Fig. S7).

A combination of Pypolca-careful and Polypolish depending on estimated short-read depth has been implemented in our automated assembly tool Hybracter from v0.7.0 [15]. Depth is estimated inside Hybracter with Seqkit [23,24] based on the chromosome size parameter estimate provided by the user. Consistent with these results, when the automated ONT-only assembly did not have structural errors [25], running Hybracter on the benchmarked genomes was consistently able to produce polished assemblies that were error-free above 25× depth, and sometimes at lower depth (Fig. S8). Short-read polishing almost always improved Hybracter assemblies. However, improvements in assembly quality were frequently not reflected in higher reference-free ALE scores [26], suggesting that when near-perfect ONT-only assemblies are polished as in this study, ALE scores have limited utility (Fig. S9).

We binned all intervals for every 5× depth increase for the three best performing polishers (Pypolca-careful, Polypolish-default and Polypolish-careful) and calculated the mean total remaining errors in each bin. Short-read polishing improved assemblies for every short-read depth bin (Table 2). Most of the benefit of polishing is seen at depths below 25×, after which the benefits of extra depth on polishing performance are minor.

**Table 2.** Mean remaining errors at all depth intervals within every 5× depth bin from 0.1× to 50× for the three best polishers (Polypolish-careful, Polypolish-default, Pypolca-careful)

Polisher	0.1–5×	5.1–10×	10.1–15×	15.1–20×	20.1–25×	25.1–30×	30.1–35×	35.1–40×	40.1–45×	45.1–50×
Polypolish-careful	33.48	20.10	13.70	11.52	8.98	8.56	7.66	7.28	6.50	6.44
Polypolish-default	34.26	24.12	15.68	11.98	8.86	8.46	7.50	7.14	6.28	6.32
Pypolca-careful	31.90	15.76	8.50	5.02	3.60	1.96	1.94	1.80	1.16	1.24

### Optimized parameters for low-depth polishing

As the default polishing parameters for tested tools are probably suboptimal for low-depth polishing, we conducted a parameter sweep on each tool to investigate whether careful selection of parameters may improve performance at low depths (Table S6). The best parameter choices for FMLRC2 (26 errors with optimized parameters vs 2514 errors with default parameters) and Pilon (19 optimized vs 778 default) had the largest decrease in error count, i.e. they benefitted the most from parameter tuning for low-depth polishing. HyPo had the smallest relative decrease in total errors (102 optimized vs 105 default), i.e. it benefitted the least from parameter tuning. While NextPolish could be tuned to have 38 % fewer errors (596 optimized vs 960 default), it still performed poorly at 5× depth, leading to a large total error count. Pypolca (14 optimized vs 63 default) and Polypolish (17 optimized vs 31 default) remained the best performing polishers after parameter tuning.

### Low-quality draft assemblies

Our low-quality draft assemblies (generated with fast-basecalled reads) had a combined total of 26 185 errors across the nine genomes. With these assemblies as input, no tools were able to fix all errors in any interval (Fig. S10). Due to the much higher initial error count, the effect of introduced errors was less pronounced than in our main analysis. However, NextPolish and FMLRC2 still increased total error counts at low depths. At sufficient depths, FMLRC2 was the best performing polisher overall (65 median errors in intervals from 25 to 50× depth).

## Discussion

In this study, we show that at low depths (<25×), many widely used short-read polishing tools frequently introduce errors in bacterial genome assemblies. The exceptions are Polypolish-careful, which never decreased the overall accuracy of any sample, Polypolish-default and Pypolca-careful, which rarely decreased overall accuracy (51/4500 samples and 147/4500 samples, respectively). We also show in this study that most of the utility in short-read polishing occurs by 25× average read depth across the genome. While the effects of introduced errors are less prominent in low-quality draft assemblies, we also found that some polishers can decrease overall accuracy at very low depths even in assemblies with thousands of errors.

While it would be unusual to have less than 25× average short-read depth across the genome for single isolate genome assemblies, there are scenarios where this concern applies even when overall sequencing depth is high, necessitating the use of conservative polishers. The first is for genomes, or regions within genomes, that have extreme GC content. It has been shown that extremes in GC content can cause issues in PCR amplification that lead to low read depth in GC-rich regions with short-read sequencing [27,28]. In such regions, short-read polishers that are not conservative may introduce errors even where the overall genome coverage is high. Local regions of low read depth may also arise in genomes with neutral GC content, particularly when transposase-based library preparation kits are used [29].

Another scenario is polishing ONT metagenome assemblies [30]. Assembling metagenome assembled genomes (MAGs) is a difficult problem due to variations in sequencing depth and community composition [31,32]. While ONT-only metagenome assemblies are improving [30], short-read polishing is still commonly recommended to improve genome completeness and protein-coding annotation of MAGs and contigs [33,34]. As even deep sequencing can often fail to recover the full extent of diversity [35], many metagenome contigs will not constitute complete genomes and have low coverage (under 25×). If these contigs are polished with short-reads with similarly low coverage, short-read polishers may introduce errors. Additionally, long-read and short-read methods may not recover the same populations from within a metagenome [34], further reducing coverage and emphasizing the need to use a conservative short-read polisher. To reduce the risk of false-positive changes, we recommend only using Polypolish-careful (the most conservative tool in this study) for polishing metagenomes, though specific studies are needed in this area.

We also showed that it is possible to optimize the parameters of most polishers to improve their performance in a low-depth context, though Pypolca and Polypolish still performed the best. Parameter optimization may be useful in extending existing polishing tools into new contexts such as metagenomes. However, applying such an approach to *de novo* sequencing is challenging because the ground truth genome is not known in advance. Additionally, the optimal parameters for one genome and read set may not be optimal for other genomes and read sets.

Overall, our analysis showed that Pypolca-careful was the single best polisher tested, with no improvements when combined with the next best short-read polisher (Polypolish-careful) in our benchmarked dataset. Therefore, for bacterial isolate assemblies from modern ONT data, we recommend short-read polishing with Pypolca-careful in all scenarios other than where depth is very low (<5×) or if avoiding false-positive changes is vital. When depth is very low (<5×) or if minimizing the false-positive polishing rate is important, we recommend exclusively using Polypolish-careful, as it only introduced a single false-positive change in the 4500 samples tested in this study.

At low depths (5–25×), we recommend Polypolish-careful combined with Pypolca-careful. Though our benchmarking did not show any improvements of this combination compared to just running Pypolca-careful (Table S5), Polypolish-careful never made an assembly worse. In other datasets, this combination could show some improvements compared to Pypolca-careful alone. At higher depths (above 25×), we recommend Polypolish-default combined with Pypolca-careful. While this was no better than running Pypolca-careful in this dataset (Table S5), adding Polypolish-default enables the polishing of errors in repeat regions that Pypolca-careful could miss (Fig. S1). We have incorporated the above recommendations into Hybracter’s [15] polishing logic from v0.7.0 and show that is it possible to consistently recover automated perfect genome assemblies when short-read depth is 25× or higher (Fig. S8).

If high-depth short-reads are available and manual curation by inspecting read alignments with IGV [36] is possible, users can try running Pypolca with relaxed settings (e.g. --min_ratio 1.5) to fix errors where short reads are inconsistent. Additionally, FMLRC2’s alignment-free approach may be able to polish errors that are difficult to fix with alignment-based approaches (Figs S1 and S10), but manual curation should be used to screen for false positive changes.

## Conclusion

In this study, we introduce Pypolca (both -default and -careful) and Polypolish-careful as short-read polishing tools. By testing a panel of nine bacterial genomes assembled from ONT reads, we show that short-read depth has a significant impact on short-read polishing performance. All polishers other than Pypolca-careful, Polypolish-default and Polypolish-careful introduced many false-positive errors at low depths. We show that Pypolca-careful is the best polisher overall and that Polypolish-careful almost never introduces false-positive errors. We recommend targeting a short-read sequencing depth of 25× or greater when creating bacterial genome assemblies. Further studies are required to assess the effect of short-read depth on polishing in other contexts, such as extreme GC content, metagenomes or eukaryotic genomes.

## supplementary material

10.1099/mgen.0.001254 Uncited Supplementary Material 1.

10.1099/mgen.0.001254 Uncited Table S1.

## Abbreviations

ALE: Assembly Likelihood Evaluation
MAG: metagenome assembled genome
ONT: Oxford Nanopore Technologies
