# Supplementary material for: How low can you go? Short-read polishing of Oxford Nanopore bacterial genome assemblies
Source: Microb Genom. 2024 Jun 4;10(6):001254. doi: 10.1099/mgen.0.001254 (PMC11261834; doi:10.1099/mgen.0.001254)
Supplement: Uncited Supplementary Material 1. [file mgen-10-01254-s001.pdf]

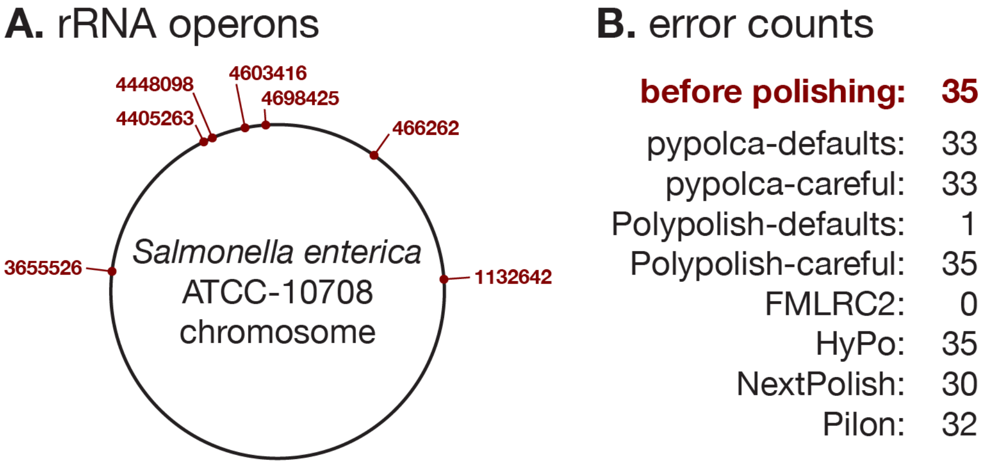

Figure S1: Polypolish-default can fix errors in repeats other alignment-based tools cannot. There was only one error out of 37 from the benchmarked Trycycler assemblies near a repeat region (*C. lari* at position 491989) and that error was at the edge of a repeat. **(A)** To illustrate the utility of Polypolish-default in repeat regions, we added five random substitution errors to each of the seven copies of the ~5.5kbp rRNA operon in *S. enterica* ATCC 10708. This created a total of 35 simulated errors per genome. **(B)** Each polishing tool was then run on the error-containing genome. Polypolish-default was the only alignment-based tool that could correct most errors, with only 1/35 remaining. All other alignment-based polishers did not correct many errors (30–35 remaining errors), although FMLRC2, a non-alignment-based method, could correct every error.

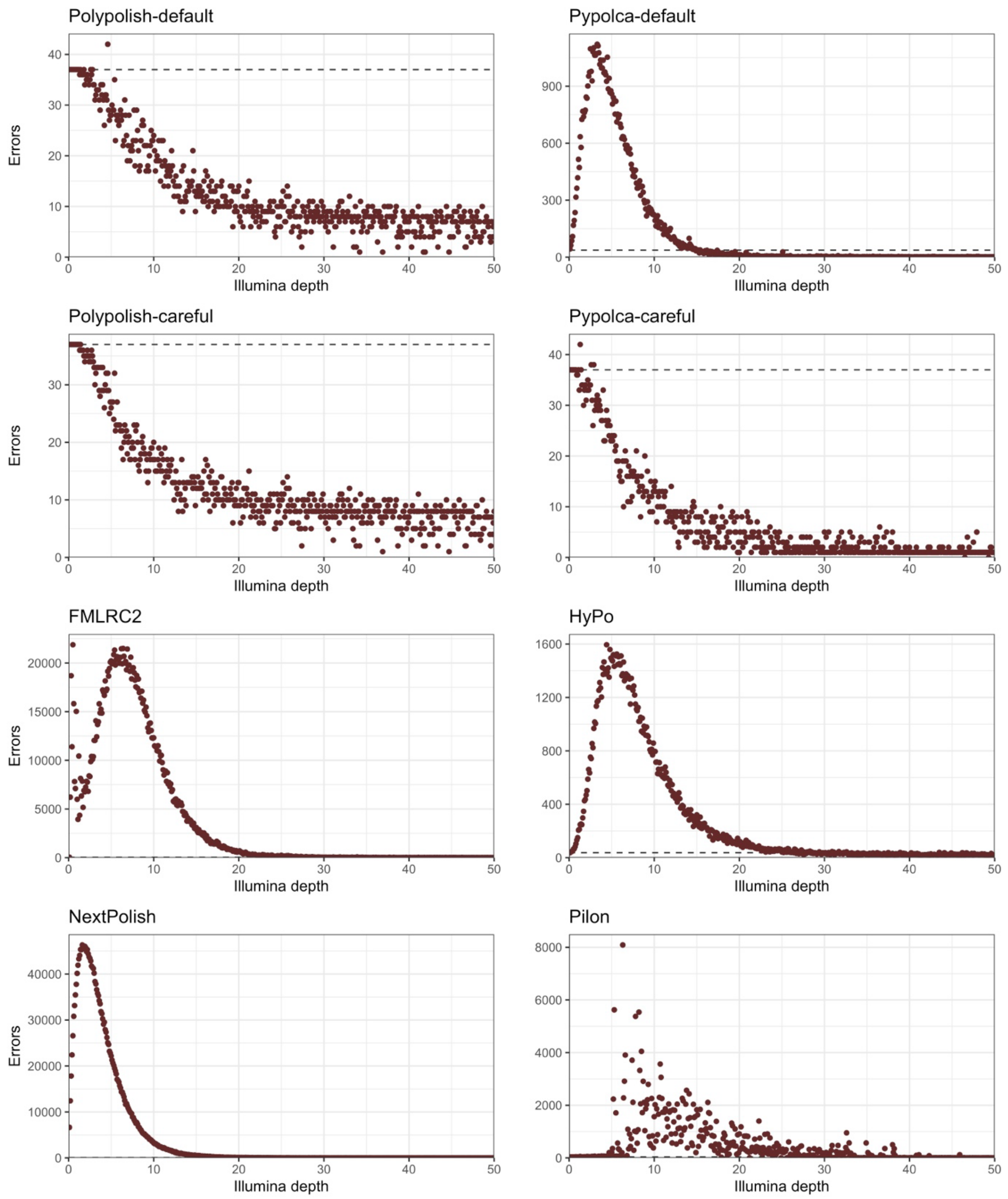

Figure S2: total errors by depth per polisher with unrestricted y-axes. This figure contains the same data as Figure 2 but with full range on the y-axes.

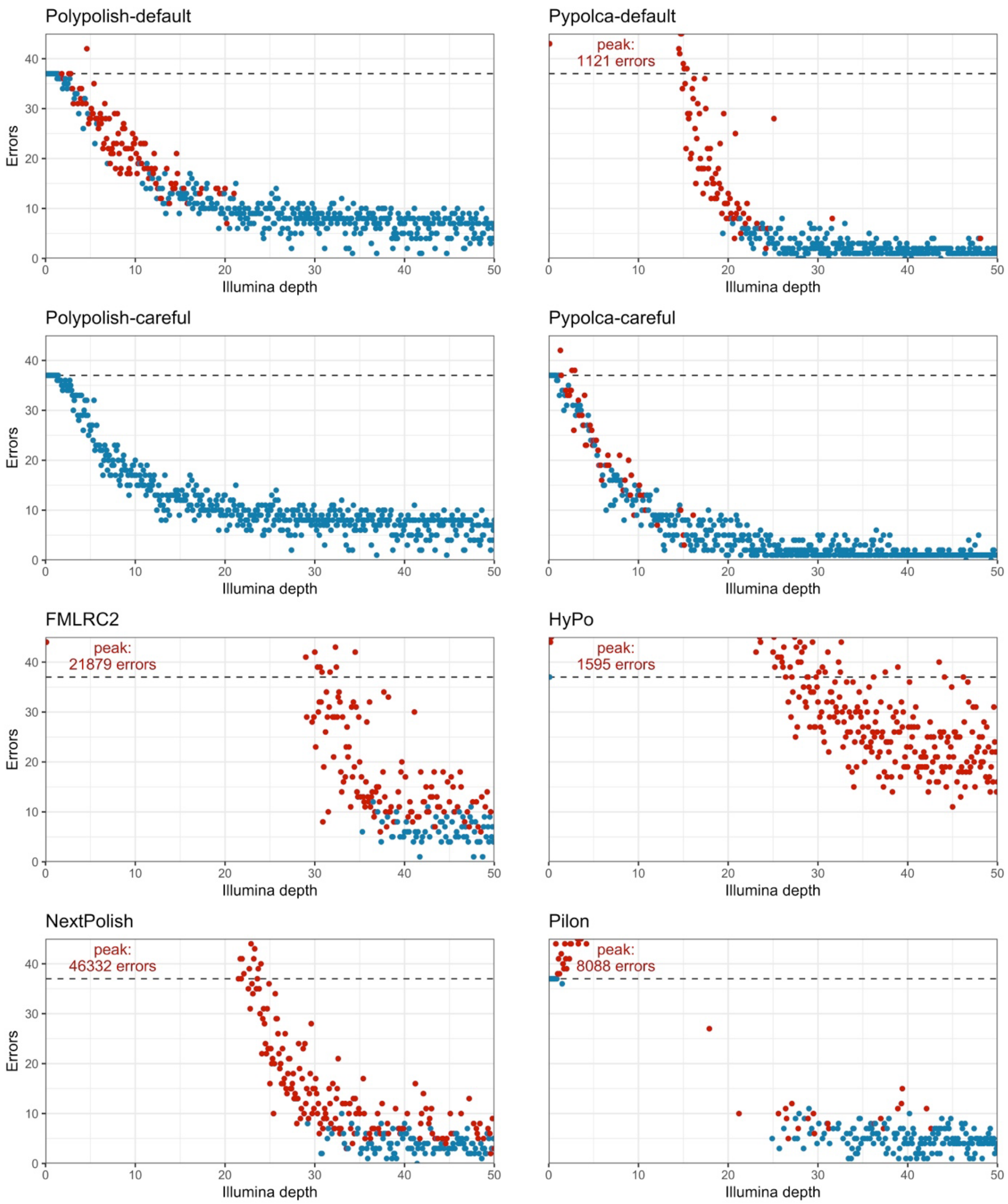

Figure S3: total errors by depth per polisher coloured where accuracy is decreased in at least one genome. This figure is the same as Figure 2 but with additional information shown in the colour of the points. Red points indicate all intervals where there were more total errors compared to the Trycycler baseline in at least one out of the nine genomes tested. Blue points indicate intervals where there all nine polished genomes had equal or fewer errors than the Trycycler assemblies.

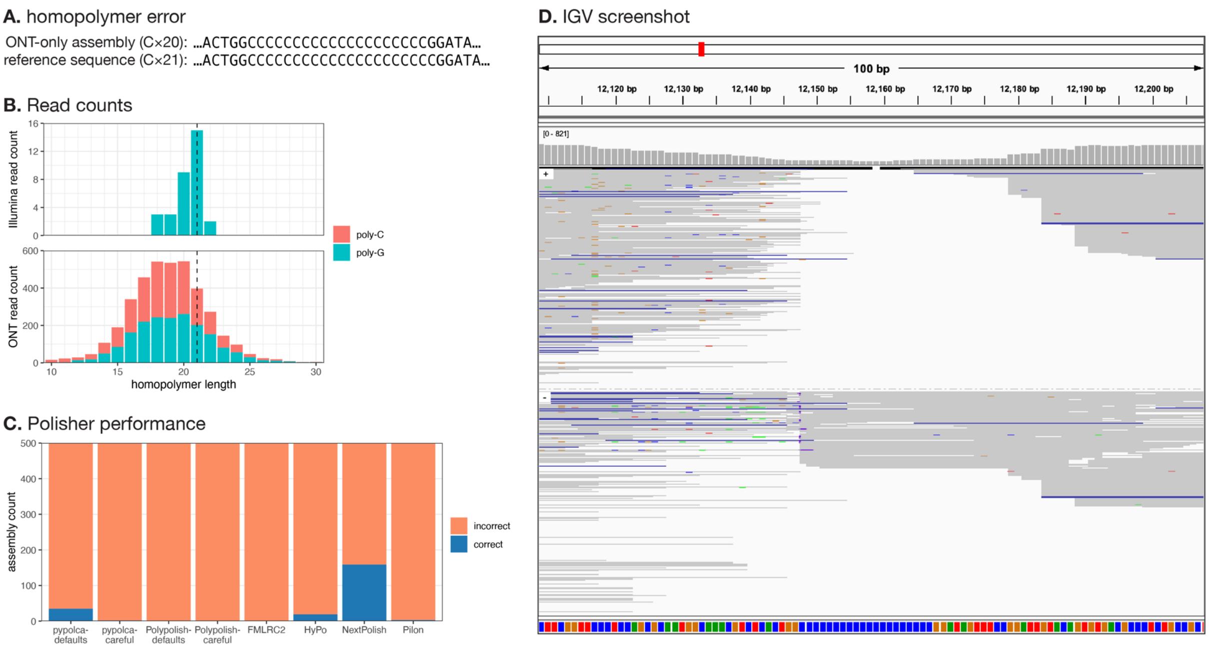

Figure S4: **long homopolymer in *Salmonella enterica* ATCC-10708. (A)** This genome's plasmid contains a long homopolymer at position 12148, and there is a discrepancy between the ONT-only assembly (C×20) and the polished reference sequence (C×21). **(B)** Illumina and ONT reads counts with exact matches to the homopolymer plus five bp of adjacent sequence on both sides. The dashed line at C×21 indicates the homopolymer length in the reference sequence. For Illumina reads, there were 32 matching reads, C×21 was the most common homopolymer length, and all matching reads were on the G-strand. For ONT reads, there were 3895 matching reads, C×18 to C×20 were the most common homopolymer lengths, and matching reads occurred on both strands. These results create uncertainty in the true homopolymer length for this genome, and the C×21 in the reference sequence is only a best guess. **(C)** Assuming that C×21 is the correct homopolymer length, this plot shows assembly counts with the correct (blue) and incorrect (orange) length at this homopolymer, for each polisher tested. NextPolish performed best, but none of the polishers were able to reliably fix this error. **(D)** Integrative Genomics Viewer (IGV) screenshot of the relevant region of the plasmid, with Illumina read alignments grouped by strand. This was generated using the full set of Illumina reads (367× depth).

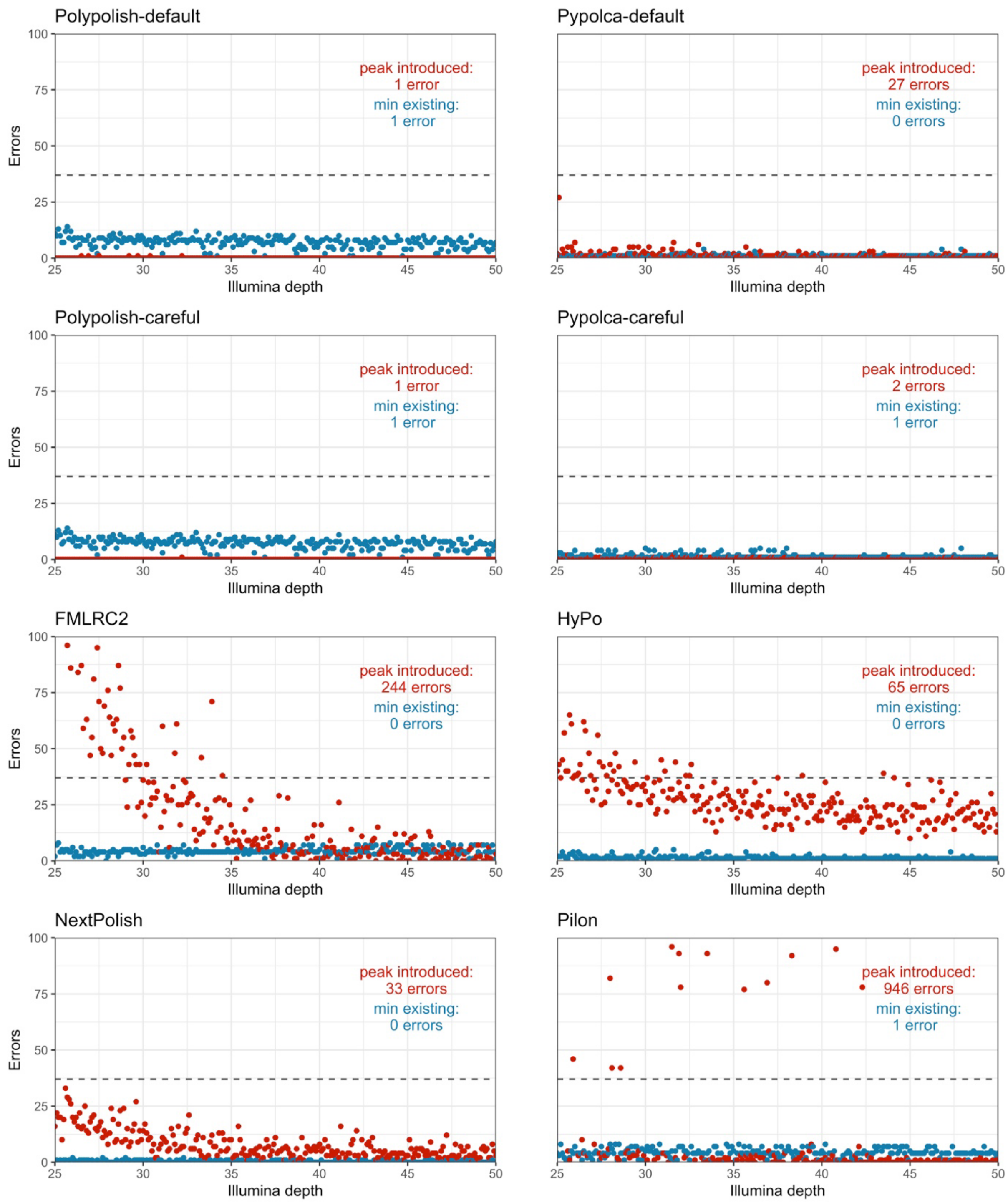

Figure S5: total introduced and existing errors by depth (25–50×). Each plot shows the total number of introduced (red) and existing (blue) errors remaining in the nine reference genomes at each sufficient depth interval from 25–50× (x-axis) for each polisher tested. The dashed lines represent the total Trycycler ONT-only assembly error count of 37 errors. Existing errors consist of the original 37 errors, while introduced errors are introduced by polishing. The y-axis is limited at 100 errors for visualisation clarity: FMLRC2 and Pilon have error counts that exceed this maximum error count.

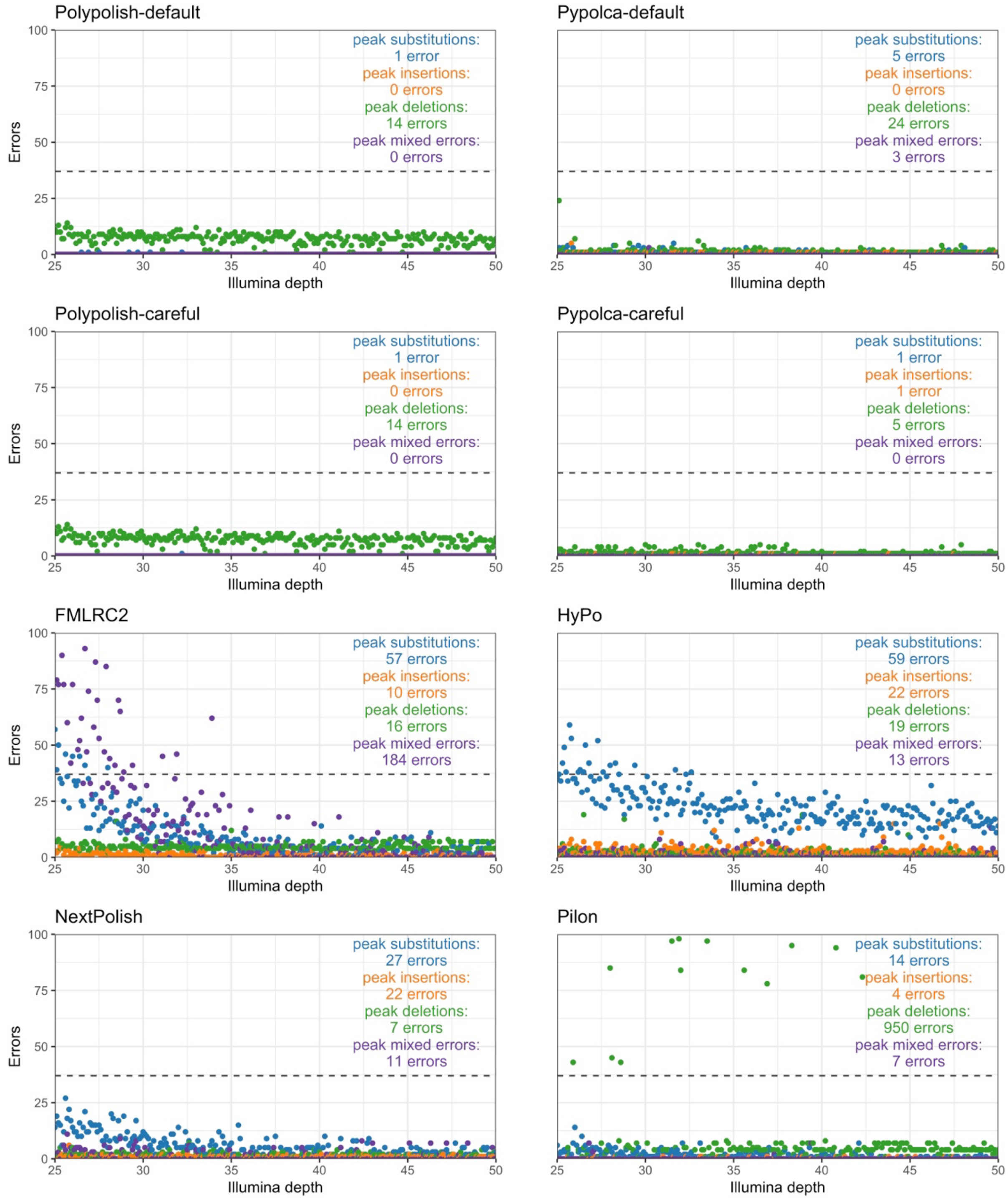

Figure S6: total substitution, insertion, deletion and mixed errors by depth (25–50×). Each plot shows the total number of substitutions (blue), insertions (orange), deletions (green) and mixed errors (purple) remaining in the nine reference genomes at each sufficient depth interval from 25–50× (x-axis) for each polisher tested. Mixed errors indicate errors where there is a mixture of substitutions, insertions and/or deletions in the same genomic region. The dashed lines represent the total Trycycler ONT-only assembly error count of 37 errors. The y-axis is limited at 100 errors for visualisation clarity: FMLRC2 and Pilon have error counts that exceed this maximum.

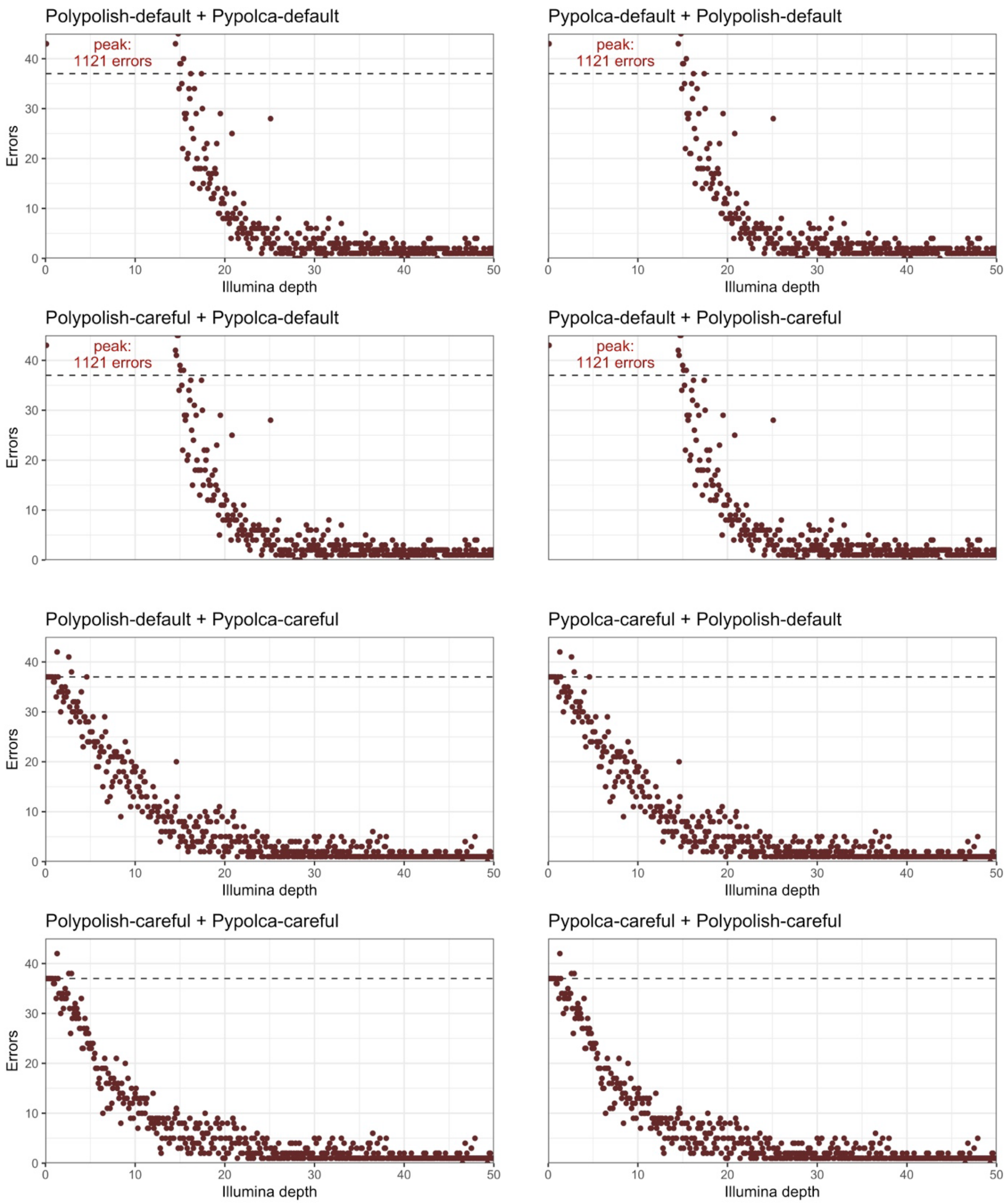

Figure S7: total errors in Polypolish + Pypolca combinations. Each plot shows the total number errors remaining in the nine reference genomes at each interval from 0.1–50× depth (x-axis) for the eight sequential combinations of Pypolca-default, Pypolca-careful, Polypolish-default and Polypolish-careful tested. The first polisher is presented first in each panel's title, followed by the second polisher. The dashed lines represent the total Trycycler ONT-only assembly error count of 37 errors. Points above this indicate that the polisher decreased accuracy and below that the polisher increased accuracy. The y-axes for the plots are limited at 45 errors, with the peak error count labelled in the top left if it exceeds 45.

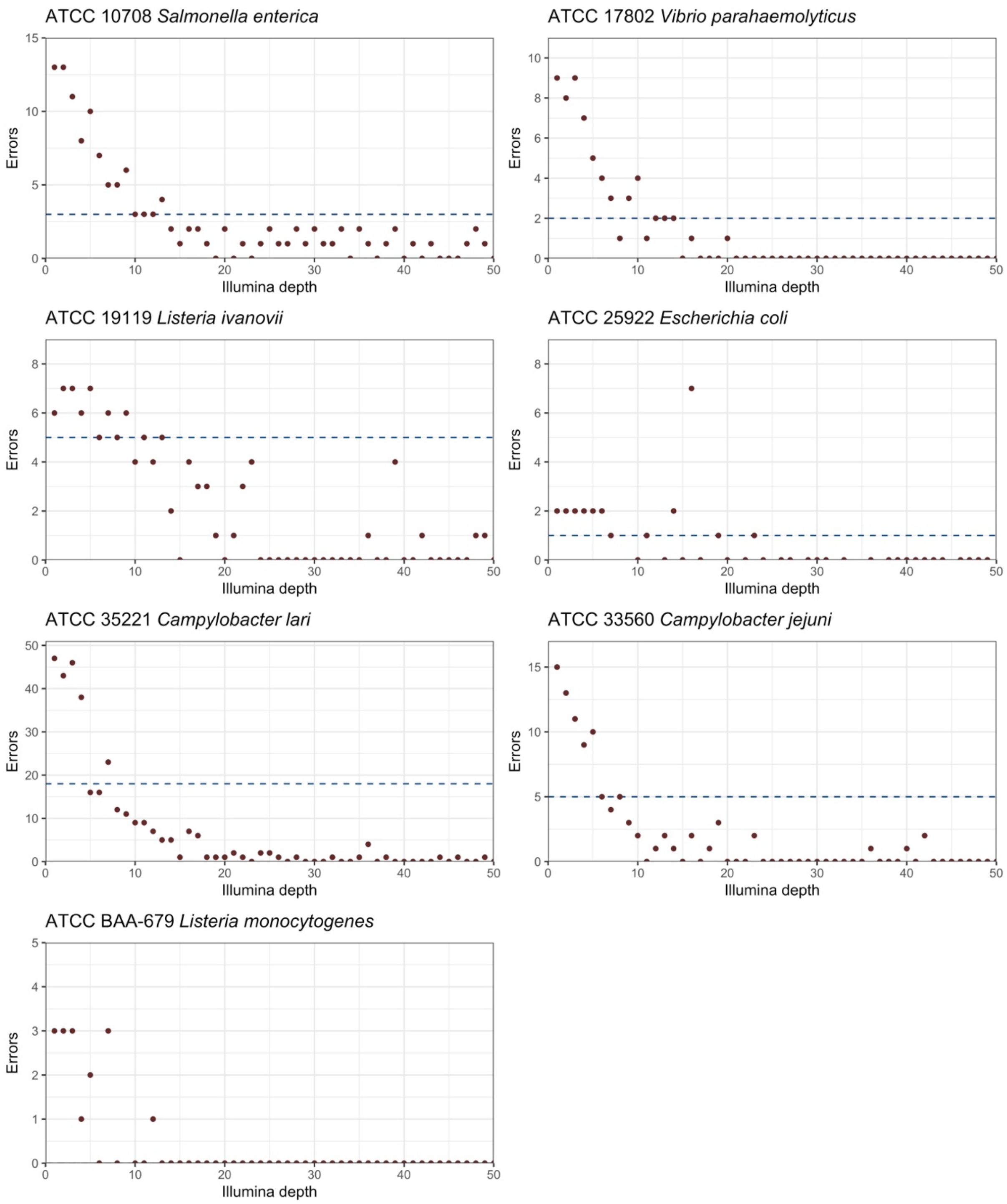

Figure S8: Hybracter v0.7.0 with short-read polishing errors per genome. For each of the seven reference genomes without significant ambiguity or non-determinism in their automated long-read assemblies, this figure shows the number of errors remaining after assembly with Hybracter v0.7.0. Each genome was tested at each interval from 1–50× depth (x-axis). The dashed lines represent the Trycycler ONT-only assembly error count for each genome, with ATCC BAA-679 *L. monocytogenes* having 0 errors in its Trycycler ONT-only assembly. Points above this indicate that Hybracter with short-read polishing performs worse than the ONT-only Trycycler assemblies, while below that Hybracter has performed better.

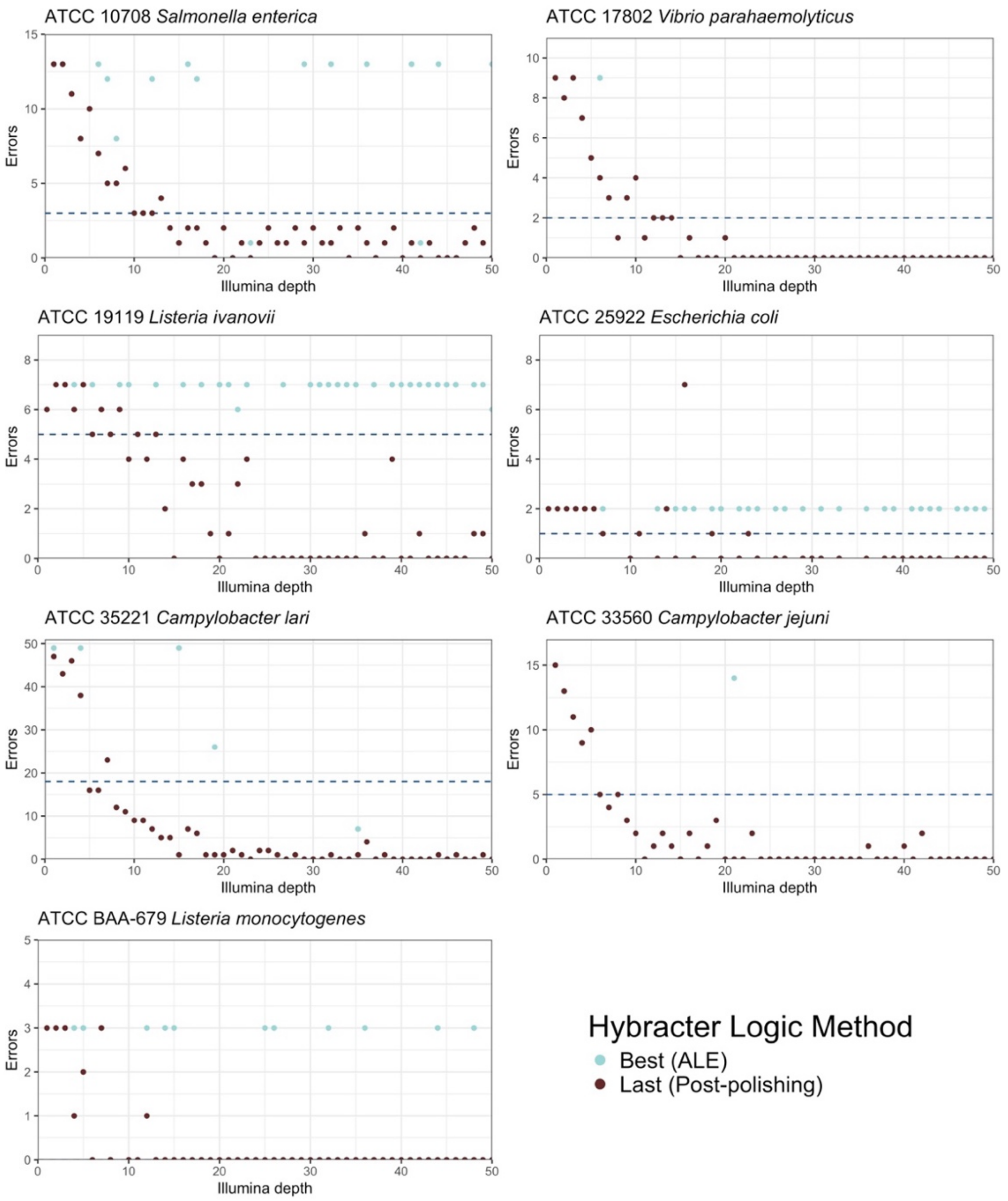

Figure S9: Hybracter v0.7.0 with short-read polishing errors per genome chosen by ALE score. For each of the seven reference genomes without significant ambiguity or non-determinism in their automated long-read assemblies, this figure shows the number of errors remaining after assembly with Hybracter v0.7.0 using the parameters '--logic best' or '--logic last'. Each genome was tested at each interval from 1–50× depth (x-axis). The dashed lines represent the Trycycler ONT-only assembly error count for each genome, with ATCC BAA-679 *L. monocytogenes* having 0 errors in its Trycycler ONT-only assembly. All blue points with '--logic best' are where Hybracter takes the polishing round with the highest ALE score as the 'best' final assembly. All maroon points with '--logic last' take the final short-read polishing round as the best final assembly regardless of ALE score. If best and last methods provide identical results, only the maroon last points are plotted. As can be seen, the best ALE score polishing round never outperforms taking the final polishing round for any genome other than one instance in *E. coli* (at 16×), and for some genomes (*L. monocytogenes*, *L. ivanovii*, *E. coli* and *S. enterica*) is frequently worse. From v0.7.0 Hybracter therefore implements '--logic last' by default and deprecates '--logic best'.

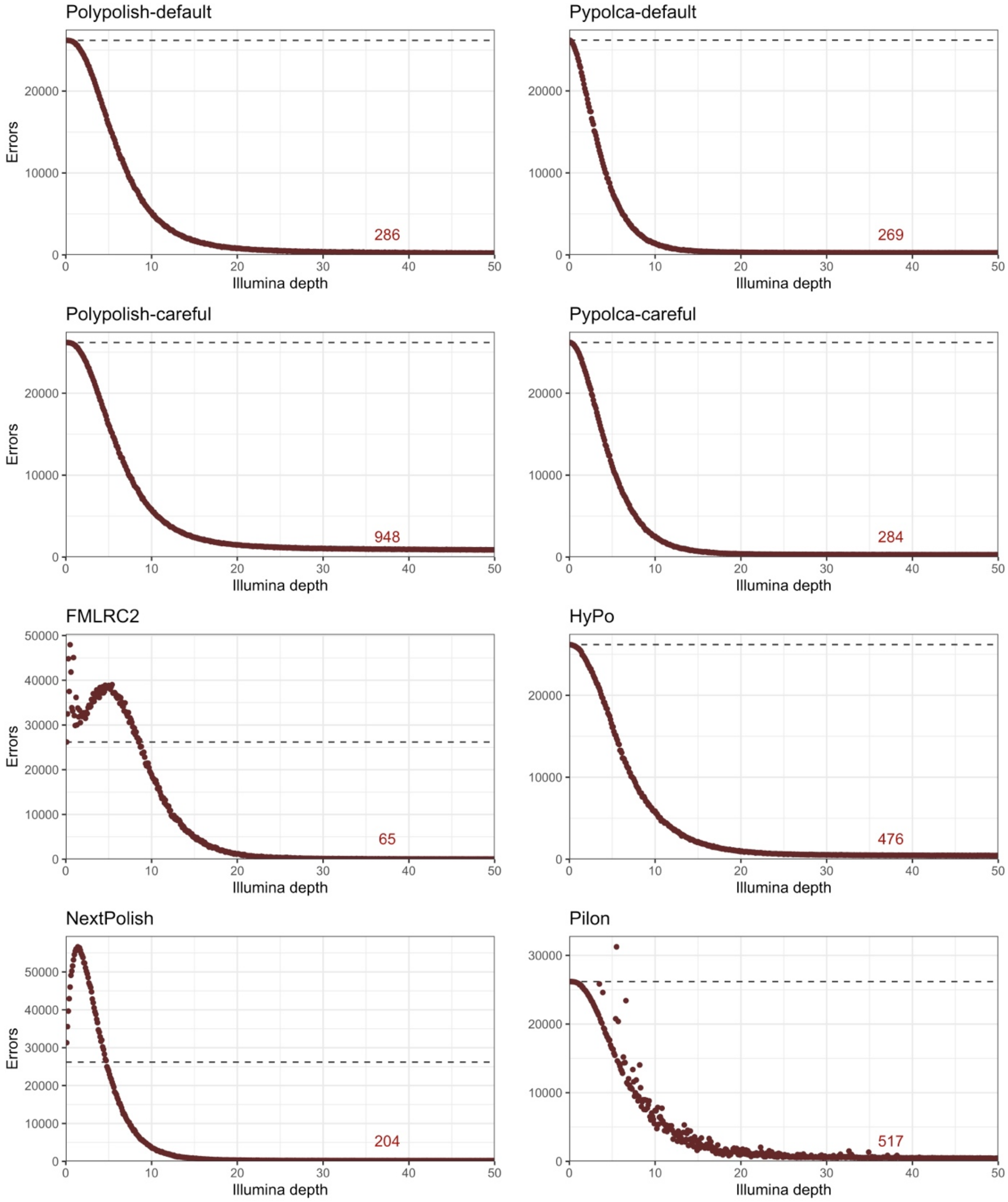

Figure S10: total errors by depth per polisher for low quality (~Q30) draft assemblies. Each plot shows the total number errors remaining compared to the nine reference genomes at each interval from 0.1–50× depth (x-axis) for each polisher tested on the low-quality draft assemblies made using fast-basecalled reads. The dashed lines represent the total Trycycler ONT-only assembly error count of 26185 errors. Points above this indicate that the polisher decreased accuracy, below that the polisher increased accuracy. The median number of remaining errors between 25–50× depth is indicated in the bottom-right of each panel.
